# A Focused Ethnographic Study of Alberta Cattle Veterinarians’ Decision Making about Diagnostic Laboratory Submissions and Perceptions of Surveillance Programs

**DOI:** 10.1371/journal.pone.0064811

**Published:** 2013-05-31

**Authors:** Kate Sawford, Ardene Robinson Vollman, Craig Stephen

**Affiliations:** 1 Farm Animal & Veterinary Public Health, Faculty of Veterinary Science, University of Sydney, Camden, New South Wales, Australia; 2 Department of Ecosystem and Public Health, Faculty of Veterinary Medicine, University of Calgary, Calgary, Alberta, Canada; 3 Department of Medical Sciences, Faculty of Medicine, University of Calgary, Calgary, Alberta, Canada; 4 Department of Community Health Sciences, Faculty of Medicine, University of Calgary, Calgary, Alberta, Canada; INRA, France

## Abstract

The animal and public health communities need to address the challenge posed by zoonotic emerging infectious diseases. To minimize the impacts of future events, animal disease surveillance will need to enable prompt event detection and response. Diagnostic laboratory-based surveillance systems targeting domestic animals depend in large part on private veterinarians to submit samples from cases to a laboratory. In contexts where pre-diagnostic laboratory surveillance systems have been implemented, this group of veterinarians is often asked to input data. This scenario holds true in Alberta where private cattle veterinarians have been asked to participate in the Alberta Veterinary Surveillance Network-Veterinary Practice Surveillance, a platform to which pre-diagnostic disease and non-disease case data are submitted. Consequently, understanding the factors that influence these veterinarians to submit cases to a laboratory and the complex of factors that affect their participation in surveillance programs is foundational to interpreting disease patterns reported by laboratories and engaging veterinarians in surveillance. A focused ethnographic study was conducted with ten cattle veterinarians in Alberta. Individual in-depth interviews with participants were recorded and transcribed to enable thematic analysis. Laboratory submissions were biased toward outbreaks of unknown cause, cases with unusual mortality rates, and issues with potential herd-level implications. Decreasing cattle value and government support for laboratory testing have contributed to fewer submissions over time. Participants were willing participants in surveillance, though government support and collaboration were necessary. Changes in the beef industry and veterinary profession, as well as cattle producers themselves, present both challenges and opportunities in surveillance.

## Introduction

In recent years, the global public health community has seen an increase in the number of emerging infectious disease (EID) events [1], with the majority of infectious agents originating in animals [2]–[4]. Countries and communities have failed to predict specific EID events and in many cases have been ill equipped to respond once a disease has emerged, making it difficult to contain both the disease and the social and environmental impacts of the disease [5]. In response to the challenge posed by EIDs, surveillance of animal populations is changing rapidly [5]. It is strongly believed that preventing or controlling disease in animals is optimal for limiting the impact of zoonotic EIDs [6], [7].

Traditional methods of infectious disease surveillance in animal health have revolved around laboratories to which samples are submitted for diagnostics, most often from clinical cases, in hopes that an etiologic diagnosis can be made [8]. Surveillance of submissions to diagnostic laboratories will continue to be an important component of any infectious disease surveillance system because for many infectious diseases laboratory tests are the only way to make an etiologic diagnosis. In addition, etiological diagnoses can inform control procedures and response policies. However, the contribution of diagnostic laboratory-based surveillance to early detection of EIDs is compromised by the time lag between the onset of clinical signs and when an etiologic diagnosis is made and the availability of diagnostic laboratory tests to identify the infectious disease agent [9]. In addition, submission biases restrict the type and number of potentially infectious cases that are submitted to a diagnostic laboratory [10], [11]. Veterinarians play a critical role in determining which cases will be submitted for diagnostic laboratory testing. Their decisions, in combination with direction from animal owners, influence the types and amounts of samples that are assessed at the level of the diagnostic laboratory, introducing potential sampling biases that will affect disease patterns described by laboratory-based surveillance [12]. In order to understand this selection bias in diagnostic laboratory-based surveillance, submission patterns of veterinarians and the factors that influence their decision to submit samples must be better understood [11]–[13].

In Alberta, in response to the need for early detection of EID events in the animal population, the Ministry of Agriculture and Rural Development (ARD) has developed the Alberta Veterinary Surveillance Network (AVSN), a multifaceted surveillance program that enables producers, veterinarians in clinical practice, and animal health authorities to respond to disease issues in the domestic animal population [14]. One component of the program is the Alberta Veterinary Surveillance Network-Veterinary Practice Surveillance (AVSN-VPS), a secure internet-based platform that allows cattle veterinarians to submit pre-diagnostic disease and non-disease case data to a centralized system. The AVSN-VPS is considered integral to the AVSN as it informs the activities of other program components, including disease investigations by ARD pathologists, epidemiologists, and veterinarians.

The success of the AVSN-VPS is dependent upon ongoing participation by private cattle veterinarians in Alberta. It began in 2005 with approximately twenty five veterinarians, and at the time this research was undertaken the AVSN-VPS covered greater than fifty percent of Alberta dairy cattle, thirty-five percent of cattle on cow-calf operations, and twenty-five percent of feedlot cattle (J. Berezowski, personal communication). Veterinarians receive monetary compensation for submissions that are received by the AVSN in a timely fashion and participation is voluntary (J. Berezowski, personal communication). In order for methods that rely on data inputs from private veterinarians to improve, continued involvement by these individuals is essential. The factors that inspired these practitioners to become involved in the AVSN-VPS are unclear, as are the reasons for ongoing involvement.

Qualitative research provides insight into human decisions and behaviour [15]. Qualitative approaches, one of which is focused ethnography, are not intended to permit researchers to make any statistical inferences from their findings that are generalized to the wider population. Instead, they allow researchers to gain a deeper understanding of the role that beliefs, circumstances, motivations, and context play in a variety of human behaviours, including decision making [15]. In other words, the strength of qualitative research is its ability to help answer *why* particular behaviours occur or to describe processes as opposed to outcomes [15] and thus is well suited to providing insight into the human dimensions of surveillance. Utilization of qualitative research methods is becoming increasingly common in the animal health field [16]–[18]. They have also been employed in the human health field to explore the use of health data in public health practice, as well as factors that act to facilitate or hinder use of these data [19]–[21]. However, in the animal and human health fields, qualitative studies are rare in comparison to the frequency of quantitative studies. The value of employing qualitative methods in understanding the human dimensions of diagnostic laboratory case submissions and participation of government veterinarians in pre-diagnostic disease surveillance initiatives has been demonstrated in Sri Lanka, a lower resource setting where the risk of disease emergence is deemed high [22]. However, Canada’s experience with highly pathogenic avian influenza, pandemic influenza virus (H1N1) 2009, bovine spongiform encephalopathy (BSE), and severe acute respiratory syndrome (SARS) highlights that EIDs are a global phenomenon [5] and understanding the ability of surveillance systems to detect and respond to EID risks in animals is necessary across a range of resource contexts.

In this paper we report the results from a focused ethnographic study that aimed to advance understanding of the factors that influence cattle veterinarians engaged in mixed-animal and exclusively cattle private veterinary practice in Alberta to submit cases to a diagnostic laboratory, and to describe the complex of factors that affect the willingness of cattle veterinarians engaged in mixed-animal and exclusively cattle private veterinary practice in Alberta who are also part of the AVSN-VPS to participate in surveillance programs.

## Methods

### Study Method

The term “focused ethnography” describes a qualitative research approach employed when what is sought is an explication of behaviour or beliefs pertaining to a specific area so that their meaning among a defined group of individuals might be understood [23]. In focused ethnography, research is not directed towards a culture but rather a particular subculture or group of participants that share some feature or features [23]. This method is used when research questions are best responded to through descriptive analysis and interpretation [23].

### Study Participants

Eligible participants were linked by their experience as cattle veterinarians in private veterinary practice in Alberta and participants in the AVSN-VPS at the time the interviews were conducted (October to December 2009). The administrator of the AVSN-VPS within the ARD initially approached participants, giving them a brief description of the research project and format and asking if they would allow their contact information to be shared with the researcher (KES).

There were only eleven prompt responses to the request for sharing of contact information and therefore the decision was made for KES to contact eligible participants as responses were received that indicated a willingness to participate. Eligible participants were characterized by sex, number of years in practice, and practice location and type. In qualitative research data saturation is defined as the completion point of the data set and results when there is data replication or redundancy, when there are no new information or themes emerging from subsequent interviews, and when the categories, themes and relationships among them are thoroughly described [24]. In studies that ask questions similar to the ones posed in this study, six in-depth interviews usually allows for data saturation, while when twelve in-depth interviews are performed data saturation is almost always attained [25]. Therefore, from the final group of fourteen eligible participants that initially responded, ten were purposively selected to take part in in-depth interviews with the aim to assemble a group of participants with maximum demographic variation in the characteristics listed previously, with an additional two selected should further in-depth interviews be required to achieve data saturation. Descriptive statistics were used to summarize the characteristics of the study participants. In order to maintain participant confidentiality, practice locations were not detailed.

### In-depth Interview Structure

The ten selected participants were contacted individually to schedule times for individual interviews. In-depth interviews were conducted at participants’ locations of choice: most often this was in their veterinary practice. While ideally all interviews would have been conducted face-to-face, three interviews were conducted over the telephone because of the long distance between KES and the three participants.

The Conjoint Faculties Research Ethics Board at the University of Calgary approved the study proposal (file number 4530). Prior to the interview, each participant reviewed and signed an informed consent form. Participants were asked at the beginning of the interview to confirm orally that they had signed the consent form. Each in-depth interview, conducted by KES, was no longer than 2 hours in length. A semi-structured format consisting of a series of three leading open-ended questions was used (Table 1). An initial set of follow-up probes was drafted and employed where appropriate: the purpose of the probes was to delve into participants’ individual responses and therefore probe inclusion and exclusion, specific wording, and order in which they were asked varied between interviews. The leading open-ended questions remained the same for each interview however the follow-up probes evolved as subsequent interviews were conducted (Table 1).

**Table 1:** Leading open-ended questions and follow-up probes used during in-depth interviews.

**Topics**	
	**Leading open-ended questions and follow-up probes**
Decision making around laboratory submissions	
	Please describe the various factors that affect your decision to submit samples for laboratory diagnostics.
	What do you see as the benefits of laboratory confirmation?
	What are the costs, in addition to monetary, of sample submission?
	Are there instances where laboratory testing is more warranted – or less warranted?
	When it comes to sample submission, who is the primary decision maker in the process?
	What kind of value does laboratory testing provide?
	Are there types of cases in which you feel laboratory testing is more urgent?
	Do you have particular ‘flags’, ‘indicators’, or scenarios that prompt you to consider laboratory testing more carefully?
Participation in disease monitoring and surveillance	
	Please talk to me about how willing you think veterinarians are or would be to participate in a disease monitoring and surveillance program.
	Why have you chosen to participate in the AVSN? Similarly, the BSE surveillance program?
	What are the obstacles to participation?
	What are the potential benefits to participation?
	Is there conflict between the different roles veterinarians are supposed to play and the interests they are compelled to adhere to or represent?
	How could veterinarians be better engaged in disease monitoring and surveillance?
	Do you think veterinarians have additional information to provide that may be missed by diagnostic laboratory based disease monitoring and surveillance?
Disease monitoring and surveillance and client interactions	
	Do you discuss disease monitoring and surveillance with your clients?
	Please talk to me about the range of attitudes you encounter, using specific examples wherever possible.
	How do you address concerns clients have about the consequences of infectious disease identification?
	What do you see as the potential benefits to such conversations?
	What do clients see as their role in disease monitoring and surveillance or do they see themselves as having a role at all?
	How concerned about the potential for disease outbreaks do they appear?
	How do you think clients could be better engaged in disease monitoring and surveillance?

All in-depth interviews were recorded using two digital audio recorders. At the end of each interview the recordings were downloaded onto a password-protected laptop computer. Both audio files were reviewed to ensure the interview had been recorded in its entirety. One file was then sent to a professional transcriptionist who transcribed the interview verbatim. Personal identifiers were removed from the transcribed files to ensure participants’ responses remained anonymous. One of the telephone interviews, the fifth interview in the series, failed to record. The error was noted immediately following the conclusion of the interview. KES immediately updated the field notes to document all data that could be recalled from the interview to enable revision of the probes used in subsequent interviews. As a result of this occurrence only nine interview transcripts were available for analysis.

After transcription of the first two interview audio files, the data were coded by interview question using QSR International’s NVivo 9 (N9), a qualitative analysis software suite that enables researchers to organize and retrieve qualitative data, including textual material. The probes were then reviewed and revised based on analysis of the first two interviews. After the third and fourth interviews this process was repeated. The probes were reviewed and revised a third time after the fifth interview. The remaining five interviews were conducted during a three-week time period during December 2009, which did not allow for transcription of the audio files between interviews. However, field notes were reviewed after each interview and therefore informed the probes in subsequent interviews. Collection of interview data concluded after the tenth interview.

Data accumulated in addition to the in-depth interview transcripts included: memos made by KES to document decisions made in the data collection and analysis process, day-to-day activities, and any comments concerning the methodological approach; a reflective journal kept by KES further describing the research process and the researcher’s experience with participants; and field notes used to record any observational data. Memos and the reflective journal were captured directly in Microsoft Word while field notes were made directly onto the interview guide during each interview and later transcribed. All raw data and material arising from the research activity were scanned into electronic files and the original documents destroyed. A single copy of the original interview audio files was transferred onto a password-protected DVD and the original files were removed from the laptop computer. The electronic version of these materials is being stored by Craig Stephen, Principal Investigator and Doctoral Supervisor, for seven years as required by the University of Calgary’s Faculty of Medicine Research Policy Guidelines for Integrity in Scholarly Activity.

### Data Analysis

The first step in data analysis involved reading through all of the transcripts to get a sense of the data set as a whole. Thematic analysis [26] was then performed on the transcripts. During this process data were systematically organized within NVivo 9 using codes that KES inductively derived from the records. In thematic analysis, concepts are basic units of analysis whose central meaning is described in a short statement, referred to as a code. These are grouped into categories, groups of content that share common features. Similarly, categories are organized around themes. Creating themes is a way of linking underlying meanings that reoccur within categories [26]. All data presented in the results section reflect the observations, insights, and opinions expressed by participants.

## Results

### Study Participants

Study participants were located in a variety of practice settings in all areas of the province of Alberta. Each participant came from a different veterinary practice; two participants were female (20%). Veterinarians had from two to 38 years (median, 24 years; mean, 22) of clinical experience. Nine (90%) veterinarians were in mixed-animal practices, while one was exclusively in beef cattle practice. Further details on the study participants are not provided to protect their identities.

### Terminology

When the examples provided by participants during the interviews referred to a particular component of the cattle industry it was often the beef industry as opposed to the dairy industry. In Alberta, the beef industry consists primarily of three types of operations: cow-calf, backgrounding, and feedlot finishing. Typically calves are born at cow-calf operations and later sold to feedlot finishing operations to be fed to market weight. On some occasions, calves are sold to backgrounding operations where they are fed for lower growth rates before being moved to a finishing feedlot operation. Producers may either be individuals with a number of mother cows who they breed to produce calves that are then sold to backgrounding operations or feedlot finishing operations, or individuals who buy calves and feed them to a desired weight. They may also own combined operations that include cow-calf, backgrounding, and/or feedlot finishing operations. Participants used the terms ‘farmer’ and ‘producer’ interchangeably.

### Overview of the Research Aims, Themes, and Categories

One theme and five categories emerged from data analysis that are linked to the aim to advance understanding of the factors that influence cattle veterinarians engaged in mixed-animal and exclusively cattle private veterinary practice in Alberta to submit cases to a diagnostic laboratory. Two themes and eight categories emerged from data analysis that are linked to the aim to describe the complex of factors that affect the willingness of cattle veterinarians engaged in mixed-animal and exclusively cattle private veterinary practice in Alberta who are also part of the AVSN-VPS to participate in surveillance programs. Themes and categories are summarized in Table 2 and linked to the research aims of this study.

**Table 2:** Research aims linked to the themes and categories that emerged during data analysis.

**Research aims**		
	**Themes**	
		**Categories**
Advance understanding of the factors that influence cattle veterinarians engaged in mixed-animal and exclusively cattle private veterinary practice in Alberta to submit cases to a diagnostic laboratory		
	Veterinarians and diagnostic laboratory submissions	
		Factors that encouraged diagnostic laboratory submissions
		Benefits realized through diagnostic laboratory testing
		Limitations of diagnostic laboratory testing
		Economic considerations related to diagnostic laboratory submissions
		Characteristics of diagnostic laboratory submissions
Describe the complex of factors that affect the willingness of cattle veterinarians engaged in mixed-animal and exclusively cattle private veterinary practice in Alberta who are also part of the AVSN-VPS to participate in surveillance programs		
	Veterinarians and surveillance	
		Willingness to participate in surveillance initiatives
		Veterinarians ought to participate in surveillance
		Drivers for involvement in surveillance initiatives
		Gains from the involvement of veterinarians in surveillance
		Participants’ perception of the role for government in surveillance
		Participants’ perceptions of the role of surveillance
	The veterinary perspective	
		Changes to the beef industry and the veterinary profession
		Cattle producers

### Theme One: Veterinarians and Diagnostic Laboratory Submissions

There were five categories identified that relate to cattle veterinarians in Alberta and their diagnostic laboratory submissions: factors that encouraged diagnostic laboratory submissions; benefits realized through diagnostic laboratory testing; limitations of diagnostic laboratory testing; economic considerations related to diagnostic laboratory submissions; and characteristics of diagnostic laboratory submissions (Table 2).

#### Factors that encouraged diagnostic laboratory submissions

Participants reported a range of factors that encouraged them to submit cases to a diagnostic laboratory. Herd-level promoters included: outbreaks where the participant was unsure of the cause; unusual rates of mortality; and potential herd-level implications of the problem. In many instances participants wished to confirm the clinical diagnosis or know the cause of the disease. Participants targeted: particular syndromes of interest; cases with poor response to treatment or pharmaceutical produce failure; cases where results from diagnostic laboratory testing would inform clinical practice; cases where there was no diagnosis from clinical or gross post mortem examination; cases where there was a suspicion of a notifiable or reportable disease; atypical case presentations; cases where the economic consequences of disease were potentially high; cases in which there was a potential public health risk; cases involving high-value animals; bizarre cases; and insurance cases. Participants also submitted samples to a diagnostic laboratory at the request of owners/producers and in instances where it was convenient. A case condition emphasized by all participants was the importance of multiple animals affected. Participants emphasized that the decision to submit samples depended on the management context:

Some guys backgrounding cattle aren’t doing anything, so if I’ve got five or six calves out of 50 that are dying, that’s not unexpected. If I’ve got a well-vaccinated herd and good management and good mineral program and good nutrition program and I’ve got more than two or three that are sick out of 40 or 50, then I’m concerned… Better managed herds have less disease but usually those kind of people usually we do more diagnostic stuff because they want to know whereas the poorer managed ones save money on management costs so they can afford to have more losses. (Interview 6, Lines 40–47)

Participants stressed that they were more likely to pursue diagnostic laboratory testing when the results impacted case management, including one participant who stressed that diagnostic laboratory testing in beef cattle practice that did not change therapy was ‘academic’:

It depends what I’m dealing with. If there’s something that I can’t answer the question without …then I need to do this. If it’s something that is academic again, it may have some benefit or it may not and the cost is significant, then it goes back to the client to decide. … Ultimately it comes down to that, my reason for testing, is it going to change my therapy when it comes to beef. If it’s not going to change my therapy then it’s academic. (Interview 7, Lines 128–134)

#### Benefits realized through diagnostic laboratory testing

The benefits of diagnostic laboratory testing referenced by participants included: enabling a definitive or etiological diagnosis; facilitating participant learning; improving confidence; and informing cases where there were legal concerns. When participants talked generally about arriving at a definitive or etiological diagnosis, they most often referenced cases from which it would have been nice to submit samples, as opposed to particular cases from which samples were sent. On the subject of facilitating learning and building confidence, one said:

As a new grad coming out… you get a lot of that counter talk where it’s “this is what’s going on, what do I do about it” and you have no confidence because cows are hard to diagnose things in anyways… So you get talking to somebody and it could be four different things and… it would be nice to be able to confirm something… So even if you don’t see that animal the second time … you’ve got it in your memory bank that you confirmed something on the last one, right? I think that in terms of developing a rural mixed animal practitioner that is actually going to stay in rural mixed animal practice, it’s extraordinarily important to be able to have the confidence in your ability to figure out what’s going on and I think that’s a huge part of retaining vets in these types of practices. (Interview 8, Lines 71–86)

#### Limitations of diagnostic laboratory testing

Participants also talked about the limitations of diagnostic laboratory testing. They mentioned that in many cases unanswered questions remain even after diagnostic laboratory testing was completed and the time lag between when samples were sent to a diagnostic laboratory and when results were available was a limitation. Carcass and tissue sample degradation in the field presented a challenge such that by the time samples were collected they had degraded to a point where they were unsuitable for many diagnostic laboratory tests.

#### Economic considerations related to diagnostic laboratory submissions

All participants talked about economic considerations that impacted their decision to submit samples to a diagnostic laboratory, often at multiple points during the interview: diagnostic laboratory testing needed to be worthwhile from the perspective of producers; diagnostic laboratory testing was cost prohibitive for producers; and the economic reality of producers meant that in the majority of instances samples were not submitted to a diagnostic laboratory. The economics of the cattle industry made diagnostic laboratory testing cost prohibitive and translated into small numbers of diagnostic laboratory submissions.

People don’t even want an exam let alone take lab samples to send away and it’s harder and harder to get on those farms because then they’re paying you for an exam and mileage…A lot of what you see is on farm looking at the rest of the herd… If you don’t get to see what’s going on on-farm, you’re kind of treating individual animals when it [the disease] may have a herd basis… so I think we’re probably missing a fair bit. (Interview 4, Lines 25–29)

When asked about costs in addition to the monetary costs of sending samples to diagnostic laboratories, one participant replied:

There is… a social cost or a reputation cost associated with sending them. People take pride in their animals and take pride in their herds and they like to have a healthy strong vibrant herd. They don’t want to have something in there that’s going to be a concern to them, […] they don’t want to have a herd that’s going to decimate the industry and they don’t want to have a herd that they’re not proud of that they’re always looking for illness or issues - I think those are the non-monetary costs. (Interview 9, Lines 9–10)

#### Characteristics of diagnostic laboratory submissions

Participants indicated that they were submitting fewer cases to diagnostic laboratories over time. They attributed this decline to a variety of factors: as you moved along in your career as a veterinarian there were fewer things you had not seen; the value of cattle has decreased, making it more difficult to submit samples; and decreases to government support for diagnostic laboratories and a decline in access to diagnostic laboratories meant that submission patterns had become increasingly selective. Some participants provided estimates of the frequency of submissions ranging from one case out of 10 to one case out of 100.

Very, very rarely. I have not sent anything this year and we’re most of the way through the fall run. I’ve talked to lots of guys about lots of sick calves this fall and have not sent one thing in, have not done one post-mortem. (Interview 8, Lines 35–40)

Participants referred to reductions in services provided by the provincial veterinary diagnostic laboratory system and a lack of large animal clinicians at private veterinary diagnostic laboratories that led to fewer submissions to diagnostic laboratories. Many participants reported that it was the producer who was the final decision maker when it came to submitting samples to a diagnostic laboratory. In contrast two participants stated that they (veterinarians) acted as the final decision maker. A number of participants discussed the ability of veterinarians to influence the decisions made by producers.

### Theme Two: Veterinarians and Surveillance

Veterinarians and surveillance occurred as a theme in the data, around which were six categories: willingness to participate in surveillance initiatives; veterinarians ought to participate in surveillance; drivers for involvement in surveillance initiatives; gains from the involvement of veterinarians in surveillance; participants’ perceptions of the role for government in surveillance; and participants’ perceptions of the role of surveillance (Table 2).

#### Willingness to participate in surveillance initiatives

All participants expressed the belief that veterinarians were willing to participate in surveillance. However, attached to this willingness were a number of caveats: there needed to be feedback of information that had value in participants’ clinical practice; data submission could not be too time consuming; participants needed to be compensated for the time they dedicated to collecting data; the data collection process needed to be convenient; and in order to motivate ongoing involvement administrators of surveillance programs should demonstrate the relevance of the data collected. Participants cited time and effort as the costs of surveillance they incurred.

#### Veterinarians ought to participate in surveillance

Participants expressed frequently the opinion that veterinarians should take a more active role in surveillance. When asked if veterinarians should be more involved in disease monitoring and surveillance, one participant replied:

You bet… I think again it comes back to a bit of a responsibility to you as a veterinarian. I think the idea of shoot, shovel, shut up type thing is just the wrong approach to take. You can only solve the issues if you know what the issues are and … find out what it is. (Interview 7, Lines 312–316)

#### Drivers for involvement in surveillance initiatives

When asked about why they opted to participate in surveillance initiatives, including the AVSN-VPS and the BSE surveillance program in Alberta, participants cited a number of drivers behind involvement including: monetary compensation; information generated and fed back through the program; interest in surveillance; perceived value of the program; and access to additional diagnostic laboratory services. The first two drivers came up frequently across interviews. A couple of participants emphasized that while monetary compensation was important to offset the time it takes to participate, it does not serve as a motivator in and of itself. Drivers behind participation varied among veterinarians.

Money talks. […] The BSE program is a good example of that. If you pay people, the right people, the job will get done. I think you’ll get a core group of preventers doing it out of the goodness of their heart because they’re interested in it and they think it’s a good program but if you want to get more people on board… reward them economically. (Interview 3, Lines 163–167)

When participants talked about information they received through surveillance initiatives, they discussed the importance of receiving that information but a few said they did not often access the outputs from the AVSN-VPS.

#### Gains from the involvement of veterinarians in surveillance

All participants talked about gains through the involvement of veterinarians in surveillance. Participants highlighted that: the AVSN-VPS could be used to inform diagnostic laboratory-based surveillance; the AVSN-VPS received a greater number of submissions compared to diagnostic laboratories; and the AVSN-VPS was timelier in comparison to laboratory-based surveillance. A couple of participants talked about past cases where the AVSN-VPS informed diagnostic laboratory-based surveillance, but more expressed the view that they would be more engaged, and the program could be improved, if there was more diagnostic laboratory support provided through the program.

We could decide what types of animals we’re interested in monitoring… We could probably decide what clinical signs we’re interested in pursuing, whether they would be of value in helping predict zoonotic problems or whether it would just help to keep the health of the herd intact… I don’t think it would be that difficult to sit down and come up with a list and maybe even a decision tree for diagnostics that the government would subsidize. (Interview 1, Lines 65–66)

Participants felt frontline pre-diagnostic disease surveillance was vital to understanding disease trends, was essential as a marketing tool, and assisted in identification of outbreaks. The AVSN-VPS made participants aware of the regional differences in infectious disease occurrence.

I think the other thing that we fail to realize […] is how different geographically, even in Alberta, certain diseases are. […] I had no idea that *Clostridium hemolyticum* was more of an issue down there. We never had it in our area. In fact, when they told me how many cases they got, I thought they were just spoofing me […] Now you take across Canada and it’s huge, […] just the different geographic areas and what diseases they see. (Interview 2, Lines 64–68)

Participants described how surveillance influences the frequency of veterinary presence on farms, referencing the BSE surveillance program in particular.

With BSE surveillance, […] financially we benefit, but… where it’s really benefited is where we were able to go out to [farms]. In the past, a farmer loses one, […] a cow dies… He thinks it incidental, drags it in the bush and that’s the end of it. When BSE hit they wanted samples from these specific ones and the ones that died were included in that. We got out there to find out what’s going on and I really felt that we learned a lot because we could go out… In numerous cases we found issues…. We never would have had that opportunity before. It got us on the farm in a non-confrontational way. It didn’t cost the guy so he was happy to have us out. […] In some cases, okay, it’s an incidental death, don’t worry about it. He was happy because he could rest at ease… In some cases, I hate to admit this in a way, but when BSE testing came about, some of our worst clients became our best from a financial standpoint because they were the poor managers in there, the ones that lost the cows and traded cows and bought cows and did all these things but at least we were able to figure out what was going on. (Interview 2, Lines 235–236)

#### Participants’ perceptions of the role for government in surveillance

On the subject of the role of government in surveillance, all participants advocated for further support for diagnostic laboratory-based surveillance from the government. Participants frequently drew attention to the costs borne by producers.

I think that there’s a big difference in the information that we want to receive and the economics borne by the producer. […] Right now the producer pays for the investigation, he pays for the test, then he may well pay for any adverse effects on his herd, his life or his livelihood that the results may show. (Interview 9, Lines 139–141)

Participants expressed the opinion that surveillance needed to be government driven and frustration with the lack of attention and resources the government directed towards disease surveillance in the animal population.

Government is so intent on cutting costs that they’re putting their animals, their industries… The billions of dollars lost with BSE is way higher than the cost of running some extra provincial government labs. […] Our government is looking at cutting costs and providing bare necessity services and moving costs onto individuals. The individuals do not have the ability to pay for the costs of testing… Those things are going to create havoc in the industry when one of these things emerges [diseases] because we do not have a proper surveillance network in place… They talk about globalization, well globalization also means the occurrence of diseases that we would never have seen before whether it’s human diseases like SARS or whether it’s animal diseases like BSE but we have to improve and have to increase our lab availability. (Interview 9, Lines 95–96)

Problems with the existing BSE surveillance program in Alberta were highlighted.

I think part of the problem with the whole program is that it got to be in people’s heads that it was out there for compensating the farmer… for these old lame skinny cows […] They [farmers] looked at it like the government doing them a favour. Then when all these restrictions came in, it was very hard to explain to people what the actual purpose of the program was and always has been […] If [animals qualify] then great, we want to give you some compensation but that was really hard for people to take […] I’d go from doing dozens a month [BSE sample submissions] to like one every five or six months. Obviously, I understand how they [the government] want to make it appealing to the producer to participate … but I think the main purpose of the program was never brought to the forefront like it should have been and that made our jobs a lot harder when they put these restrictions in place because these people are yelling and cursing at us and you’re just trying to explain what the whole point of it is. (Interview 4, Lines 92–100)

Participants discussed their perceptions of government. The Canadian Food Inspection Agency (CFIA) was not viewed favourably, though the provincial government fared better. One participant stressed that the AVSN-VPS added to their respect for the provincial veterinarians as they saw the AVSN-VPS as a collaborative effort between veterinary practices and the province. One participant articulated dissatisfaction with the CFIA and its handling of reportable disease cases,

Reportable diseases that occur in the area the CFIA picks up, do you think we’re notified first on the list that one of our clients might have a certain problem? No. We’re usually one of the last people to find out and usually it’s from the producer. I think that’s pretty terrible […] Yeah there was one in our area from one of our clients and I knew nothing about it until he came in having all these questions… He was given very little information by them and I ended up having to phone the CFIA and chase someone down to talk to and get the story… Something reportable is right here in our own backyard and we weren’t even notified by them […] It was on a random screening sample at one of their plants and they picked it up […]

Not only is that very poor relations but it sends a bad message to us because veterinarians are proactive type “A” people that want to be involved and if I’m going to go out […] and invest my time, my effort and I care about this and … something comes back or you find something out about a herd in our area and then you don’t even bother to contact me and let me know, I think it sends a really bad message out: “We don’t want to work together. We don’t want to involve you or help you” - so that makes it difficult too when they want us to do stuff for them or send a certain message. (Interview 4, Lines 137)

Participants stressed the importance of communication, emphasizing that problems could persist if information was not made available to veterinarians and producers for use in prevention and treatment.

#### Participants’ perceptions of the role of surveillance

Participants talked about surveillance and the greater good or its value beyond infectious disease event detection. Several participants discussed surveillance outputs to inform clinical practice and increase awareness of regional differences in infectious disease burden. In contrast, one participant expressed the opinion that disease had not changed much over the past twenty years and pre-diagnostic disease surveillance programs did not help significantly in addressing infectious diseases, though such programs were great for the international reputation of the cattle industry in Alberta.

Participants cited frequently that surveillance benefitted the cattle industry, though a few expressed frustrations that producers were not deriving any benefit from increased surveillance.

I have been frustrated. With a variety of these programs we’ve done a lot of hoops and it’s just not changing this industry. It’s in a sad state and yet they’ve [producers] connected the dots that have been asked of us … You just keep on plodding hoping that at some point it will be recognized. (Interview 7, Lines 365–367)

Participants also cited veterinarians, the industry, and the public as beneficiaries of surveillance. During a number of interviews surveillance for EIDs was mentioned in particular, including one participant who expressed scepticism about the ability of the AVSN-VPS to provide information that might be missed by diagnostic laboratory-based surveillance.

### Theme Three: The Veterinary Perspective

There were two categories identified that related to the veterinary perspective: changes to the beef industry and the veterinary profession; and cattle producers (Table 2).

#### Changes to the beef industry and the veterinary profession

All participants discussed the dynamic nature of the beef industry, the veterinary profession, and the relationship between the two. Economics were often drawn into the discussion. Emphasis was placed on the need for financial compensation to motivate changes to the beef industry.

Unfortunately I think a lot of producers, they won’t change unless they have to and there’s two ways you can do that, you can force them to by saying that you have to put these tags in or you’ll get fined or we can say you have to do it or you can’t sell your product. I think probably the better way is you somehow make these subtle changes in the system… We’re starting to do that anyway but the problem is if you’re going to make those changes, you have to make it economical for the producer… You can’t continue to download […] a lot of work …and regulations on this producer and then expect him to do it and not be compensated. He’ll just get out. (Interview 3, Lines 260–262)

Participants highlighted that the role played by veterinarians had historically been different and was bound to continue to change.

I support my family by doing a lot of technical stuff… pulling calves, pushing prolapses, preg[nancy] testing cows. […]The connection between animal and human disease and looking at the big picture, that’s incredibly important and that’s going to be a sustainable aspect of our profession. I think it’s unrealistic to think that […] the next generation veterinarians are going to do what I do. I showed you rings in the back of the clinic. You know that guy obviously made a living doing a thousand Caesarians in the spring. […] He made a significant portion of income by vaccinating heifers for brucellosis. I don’t do that anymore and so why would I expect the next generation of veterinarians to do what I do for a living… What do we do as a profession to maintain our relevancy? (Interview 3, Lines 105–111)

One participant described how much the veterinary profession had changed during their career.

I mean I’ve had herds that when I first started here in ’94 that were losing ten or fifteen percent of their calf crop just with scours and through better management and vaccine programs, we’ve reduced that to less than two percent. So absolutely we make them money. […] We’ve gone past that though […] Historically that was true because we could make some big changes […] When I started 30 years ago, it was an astronomical problem with bulls and Caesarians. We were doing two to three hundred Caesarians every fall in a 5,000 mother cow practice. Now in a 5,000 mother cow practice, we might do four or five Caesarians because we’ve improved the mother cows. We’ve improved the bulls. We’ve improved the feeding programs. […] It’s much smaller [the gains that can be made] so for them to quit using veterinarians now doesn’t make as big an impact as it did before. […] With us going away, they can still buy all their vaccines… We don’t have any control of that like they do in Europe and other countries where they have to be bought through a veterinarian. (Interview 6, Lines 172–197)

The same participant predicted that cattle veterinarians in small mixed practices would no longer exist once current veterinarians retired.

#### Cattle producers

During all of the interviews the circumstances of producers was touched upon, and perceived to be as dynamic as that of veterinarians.

I think they’re in a similar position that we are, they have to change, they can’t continue to raise cattle the way their grandfather did, just like we can’t continue to practice veterinary medicine like three generations ago. Part of that education process is I can count on one hand young cow producers that want to produce cattle, the majority of guys are old or older. If you can target these young guys that are ambitious and want to do it, you have to convince them that they have to do it differently and that’s part of the education process is “how can I help you do something different to be sustainable and make a living raising cattle instead of having to have two off-farm jobs to support the farm”, and that’s a challenge. (Interview 3, Lines 237–239)

Participants expressed the view that producers feared a reportable or notifiable disease, though in contrast one participant expressed the view that producers would love it if the government were to come in and compensate them for the loss of their herd due to a notifiable disease as it would be a way for the producer to exit the cattle industry.

In the opinion of participants the fear of a reportable or notifiable disease was in part attributable to producers fearing the stigma of being the person in the community with the affected herd.

They understand that the chances of them having a positive is extremely low. What they’re scared of is being in the spotlight and all of a sudden the neighbours, you know it’s a bad stigma. […] You don’t want to be the guy that’s got a … positive anything - so I guess it’s education on our part that it’s sort of like, you know they tell people with cancer, the one thing worse than finding it is not finding it right? So you tell them that that if you don’t find it now, that you’re going to find it eventually. (Interview 3, Lines 220–223)

Some participants discussed the importance of independence to producers along with the concern that once current producers got out of cattle farming there would be no one willing to farm cattle in Alberta.

The only reason you farm is a lifestyle. I shouldn’t say the only reason. It’s one of the biggest reasons that people farm. It’s a great place to raise a family and you’re outside, you’re your own boss, nobody else telling you, you have to do this. I don’t have to get up today if I don’t want to or I can work all day if I want to, … and that has appealed to most of the people that come from a rural environment and they want to come back to that. A big chunk of my clientele […] grew up on a family farm… They work in the oil patch to support their farm, and on their holidays, they come home and make hay. Their kids resent the farm and they will not take over the farm. […] So the father who grew up feeling the farm was part of him and liked that, he comes back, can’t afford to farm but can live on a farm, have a bit of a hobby farm with oil patch industry and income. It dies with him. When he’s out of the game, there’s nobody taking it over and they’re a big chunk of who’s supplying the cattle right now. (Interview 7, Lines 406–412)

Finally, a number of participants raised confidentiality and privacy as of concern to producers. In relation to surveillance initiatives and producers, one participant said:

There’s a lot of less open minded people out that are very anti-government and there’s also just people that aren’t necessarily anti-government but that value privacy… I think if there’s a way that we could surveil more anonymously, that would be [ideal] and you know people are always more willing to accept that than if they have to put their name on something. For example, this [interview] right, if I’m going to talk … give you all these examples, I don’t want people to know I’m from {town name} or people will be like who in {town name} has this disease you know so I understand that … And some people are just very private and think whatever goes on, on my farm, is my business. (Interview 4, Lines 143–147)

Another participant expressed a slightly different view:

I think most producers want these kind of [surveillance] programs. They want to know what the diseases are in their cattle and they want to participate in making our, or making their, product healthier and better and superior to other countries. […] I don’t think there’s anybody that really wants to hide anything. I think there’s openness in most of these people, they’re not afraid to share their information with anybody. At least not my clients… I mean they don’t want us sharing it with all their neighbours, but with the government, that’s alright. (Interview 6, Lines 157–162)

Following analysis of the nine interview transcripts the codes, concepts, categories, relationships, and themes were reviewed. The authors observed that there was data redundancy and the categories, themes and relationships between them were thoroughly described. It was also noted that though the last few interviews enriched the data set, they led to no new information or themes. Therefore it was determined that data saturation had been achieved and there were no further interviews conducted.

## Discussion

### Veterinarians and Diagnostic Laboratory Submissions

Study participants detailed a variety of factors that encouraged diagnostic laboratory submissions, with multiple animals affected and the impact of results on case management common to a number of scenarios. Participants stressed that the decision to submit samples depended on the management context and the impact of results on case management. Participants detailed some of the benefits and limitations of diagnostic laboratory testing that also factored into their decision to submit samples to a laboratory. However they also reported low submission rates and submission of fewer cases to laboratories over time. Economic realities, including the high cost of diagnostics relative to the decline in value of individual beef cattle, as well as a decline in government support for laboratory diagnostics, had contributed to a decreasing frequency of laboratory submissions over time.

The results show that diagnostic laboratory submissions from participants were biased toward: outbreaks; outbreaks with unusual mortality rates; atypical case presentations; bizarre cases; and cases with poor response to treatment or produce failure. Assuming that participants’ submission patterns reflected those of cattle veterinarians in Alberta and remain relatively unchanged over time, the patterns detected by diagnostic laboratory testing are unlikely to reflect disease burden in the Alberta cattle population. This finding is supported by quantitative studies looking at diagnostic laboratory test submissions [11], [12]. Consequently the patterns of diagnoses based on diagnostic laboratory findings should not be assumed to reflect disease trends in the Alberta cattle population and it may not be appropriate to rely solely on disease prevalence outputs reported by diagnostic laboratory-based surveillance to guide future research priorities.

We recently undertook a similar research project with government field veterinarians in Sri Lanka [22]. It is interesting to note that while the circumstances of veterinarians in Sri Lanka were different to those in Alberta, there were similarities in the challenges to diagnostic laboratory testing across contexts, namely the availability of sufficiently timely results to inform treatment and access to desired diagnostic laboratory infrastructure. The outcome in both contexts was that veterinarians have become accustomed to relying on other means to make a diagnosis and guide treatment. Changes to the veterinary diagnostic laboratory infrastructure that would significantly impact this challenge to diagnostic laboratory-based surveillance would require considerable investment and political will, and the time to realization of the benefits of such efforts could be lengthy in both the Alberta and Sri Lanka context, particularly if no emerging disease issues were immediately detected.

One way of examining the diagnostic laboratory submission behaviour of participants is through the lens of expectancy theory from the field of sociology. Expectancy theory is concerned with the process individuals go through in arriving at the decision to perform one behaviour over another or others [27], [28]. At its foundation is the idea that individuals decide to act in certain ways because they are motivated to select particular behaviours out of a range of possible behaviours due to the results they expect to stem from them. There are three components of expectancy theory: expectancy, instrumentality, and valence [27], [28]. These three components play an interactive role in motivation. A large part of expectancy theory is what individuals perceive: individuals’ actions will not be motivated by what the results will be, but by what they *believe* the results will be. One of the primary goals of cattle veterinarians in private veterinary practice in Alberta is to achieve positive case outcomes for their clients. Application of expectancy theory in this context reveals that if a veterinarian perceives a strong correlation between performing diagnostic laboratory testing and case outcome then instrumentality (an individual’s belief that the rewards acquired as the result of an action are closely related to level of performance) will be high and the veterinarian will be motivated to pursue laboratory diagnostics. This theory helps to explain why diagnostic laboratory testing that does not inform treatment was viewed as ‘academic’. However, participants also cited suspicion of a reportable or notifiable disease or concern for a public health risk as case characteristics that encourage sample submission. In these instances the goal may be to confirm the absence of a reportable or notifiable disease or a public health risk. Though based on past experience the likelihood of a reportable or notifiable disease or public health risk is low, the valence (the degree to which an individual values a particular award) attached to identifying either event is high.

Participants reported that the time lag between when samples were submitted to a laboratory and when results were available had lengthened as the diagnostic laboratory infrastructure in Alberta has changed. Additionally, the decline in cattle value and government support for diagnostic laboratory testing meant that the financial burden of diagnostic laboratory testing borne by producers might have been too great a cost compared to the perceived benefits diagnostic laboratory testing provided. Participants reported getting onto farms less and less, presenting fewer opportunities to even consider submission of diagnostic laboratory samples as an option. These factors have impacted the number of opportunities for veterinarians to *perceive* the benefits of diagnostic laboratory submissions, and likely would have had the greatest impact on recently graduated veterinarians for whom diagnostic laboratory testing also facilitated learning and built confidence.

There are strengths and limitations to relying on diagnostic laboratory submissions from cattle veterinarians in Alberta for EID event detection. EID events characterized by atypical case presentations or bizarre cases are likely to make it to the level of the diagnostic laboratory, though participants reported that it would be unlikely for the index case to be submitted. Submission to diagnostic laboratories would also necessitate veterinarians to recognize that a number of cases over time were sufficiently similar to have an underlying etiology. The ordered diagnostic laboratory test would have to be capable of detecting the agent or, in the event that histopathology or cytopathology were the test ordered, the pathologist would need to recognize that the case represented something out of the ordinary. Alternatively, the diagnostician reviewing the case history would need to come to the conclusion that additional diagnostic tests were warranted, and consult with the veterinarian about additional testing and cost coverage. Participants also indicated that samples were submitted when there were unusual outbreaks or in situations where there were large numbers of animals affected. Surveillance of diagnostic laboratory submissions may therefore be sufficient for detection of EID events characterized by these types of presentations, though it is difficult to determine if detection would be sufficiently prompt to mitigate their impact on animal and public health. In contrast, given the overall small number of sample submissions reported by participants, diagnostic laboratory-based surveillance is unlikely to detect slower-moving EID events that present more sporadically or changes in trends of known endemic problems as incomplete sampling is unlikely to generate a signal in the diagnostic laboratory data stream [29].

The AVSN is part of the Canadian Animal Health Surveillance Network (CAHSN), a network of provincial, federal, university, and private animal health diagnostic laboratories [30]. This newly established network aims to: increase diagnostic laboratory capacity to detect infectious animal diseases; permit implementation of common protocols, including use of common reagents; coordinate surveillance activities; enable the sharing of technical and scientific expertise; and enable collation and analysis of laboratory data from participating diagnostic laboratories [30]. The objective of the CAHSN is “early detection of animal disease threats to the food supply, food safety or public health originating through bio-terrorism or ‘natural’ causes, especially foreign and emerging animal diseases” [30]. While this integration effort helps to ensure there is sufficient diagnostic laboratory capacity in place to respond to EID events, and detect certain types of EID events, the results reported here suggest that such efforts alone will be insufficient to permit early detection of animal disease threats: diagnostic laboratory submission results are unlikely to signal the occurrence of an EID event in the Alberta cattle population early in the epidemic process [29].

### Veterinarians and Surveillance

Participants expressed a willingness to participate in surveillance initiatives, though their involvement required support via monetary compensation, feedback of relevant data and information, demonstrated program value, and subsidized diagnostic laboratory support. Further, participants expressed the belief that veterinarians should take a more active role in surveillance. They cited information to guide laboratory-based surveillance, greater numbers of submissions, and more timely information as gains from veterinary involvement in pre-diagnostic surveillance. Participants advocated for increased government involvement in surveillance, though they stressed that efforts should be collaborative.

Animal health surveillance is undertaken by people in a wide range of contexts: the practice of surveillance is directly related to the environment in which it takes place and therefore a socio-ecological approach to analysis is warranted. There are a number of variations of the socio-ecological model that have been developed based on the work by Bronfenbrenner, 1979 [31]. They all identify levels of influence on human behaviour that overlap and taken together comprise the environment in which human behaviours take place. An assumption inherent to the socio-ecological approach generally is that assessment and approaches to intervention that operate at multiple levels are more effective in comparison to those that operate on a single level [32]. For the purpose of this paper, five levels of influence will be individually explored (individual, interpersonal, organizational, community, and societal) that are widely utilized when adopting a socio-ecological approach [31].

#### Individual-level influences on surveillance

The individual level in the socio-ecological model emphasizes the importance of characteristics of the individual to intervention strategies. Cattle veterinarians in Alberta are part of a private industry and therefore some form of compensation for time dedicated to surveillance initiatives is essential. However, animal health surveillance is not the only duty of these veterinarians: the results show that while monetary compensation was important, it was not sufficient to guarantee veterinary participation in surveillance. Participants emphasized that surveillance that relies on private clinical veterinarians to input data must generate information that is of value to veterinary clinical practice. One challenge to animal health surveillance programs is that they need to serve the interests and needs of a number of stakeholders including governments, consumers, industry stakeholders, and producers [33]. Surveillance that is dependent upon veterinarians in private practice to submit data has the additional responsibility to provide data submitters with information that is clinically relevant [33]. Future surveillance initiatives and modifications to existing programs must take this task into account during design, implementation, and evaluation to help ensure surveillance system sustainability.

#### Interpersonal-level influences on surveillance

The interpersonal level in the socio-ecological model emphasizes the importance of social norms and social influences to intervention strategies. Veterinarians have an ethical duty to promote public health defined in the veterinarian’s oath [34]. Participants expressed a willingness to contribute to pre-diagnostic surveillance initiatives, the belief that veterinarians should take a more active role in surveillance, and the opinion that government needs to deliver surveillance programs. However, the results show that this approach needs to be one of collaboration and must take into account the relationship between producers and veterinarians. The success of private veterinarians is dependent upon their relationship with producers: it is imperative that surveillance initiatives reliant on the participation of private veterinarians respect this relationship and not serve to undermine it. For example, pre-diagnostic surveillance initiatives may need to include mechanisms that ensure specific farm locations are excluded from case submissions in order to protect the privacy of producers and gain support from veterinarians, as was done with the AVSN-VPS (John Berezowski, personal communication). In addition, the goals of surveillance initiatives need to be communicated to producers so that when changes are made that are deemed necessary producers understand the reasons behind them. An even better approach would be to include producers in the process of negotiating changes to existing surveillance initiatives so their comments and perspective are considered and they are not caught off guard when changes are made.

While it is common practice to calculate the economic consequences of EIDs [35] and investigate their impact more broadly [36], projecting the economic benefits realized through surveillance remains a challenge [37]. It is also impossible to pinpoint EID events that have been averted as a result of surveillance. The results show that the BSE surveillance program in Alberta that requires veterinarians to visit cattle operations to collect samples has had both direct and indirect consequences to the veterinary perspective on the cattle health situation. While it serves to satisfy many consumers and trading partners that the prevalence of BSE in Canada’s cattle population is very low, and the risk of a BSE-positive cow entering the food chain is very small, it has also translated into more veterinary contact with the cattle population, in particular with segments of the population that previously had minimal contact with the veterinary profession. This increased contact could prove essential to recognition of future EID events. Creating circumstances for veterinarians to get onto cattle operations in the absence of a major problem, or in a ‘non-confrontational way’, has had the added benefit of improving the relationship between veterinarians and producers. This enhanced affiliation could prove invaluable during future EID events as producers might be more likely to bring animal health concerns to the attention of their veterinarian, creating more opportunities for event recognition, thereby enabling more timely EID event detection and response. It could also be critical to enabling veterinarians to influence the producer’s final decision when it comes to submitting samples to a diagnostic laboratory, thereby enabling more cases to reach the level of the diagnostic laboratory and potentially improving this source of surveillance data. Previous work has also suggested that the trust of producers is critical to event reporting, surveillance, and adoption of biosecurity measures [38], all of which are critical to EID event detection and response.

#### Organizational-level influences on surveillance

The organizational level in the socio-ecological model recognizes that changing the policies and practices of a workplace can serve to support behavioural change. In Alberta, providing the ARD with additional resources to support the activities of cattle veterinarians, in particular further diagnostic laboratory capacity, is an incentive for surveillance system participation that was identified by participants as essential. As suggested by one participant, collaboration on a list or decision tree that would inform diagnostic laboratory testing supported by the government is one approach to future diagnostic laboratory-based surveillance by the ARD that had been unexplored at the time of the interviews. This type of approach could be particularly useful as it would enable targeted case presentations to reach the level of the diagnostic laboratory and it would heighten awareness to these case presentations among cattle veterinarians. Efforts to communicate with farmers about such programs would help to ensure cases are being brought to the attention of veterinarians.

#### Community-level influences on surveillance

The community level in the socio-ecological model recognizes that coordinating the efforts of members of a community, in this case cattle veterinarians in Alberta, is necessary to bring about change. The results demonstrate how the AVSN-VPS has served to provide cattle veterinarians in Alberta with a shared perspective on the burden of clinical disease in Alberta’s cattle population, an essential first step in bringing together members of a community [39], [40]. However, the results also indicate that the information produced from the AVSN-VPS has had limited utility in cattle veterinary practice. Administrators of the AVSN-VPS should consider consulting with veterinarians who input data to determine how to make the information provided more relevant to data providers, and if any further data types might be worth collecting. This consultation process would also serve to enhance the collaboration between the AVSN and cattle veterinarians.

#### Societal-level influences on surveillance

The societal level in the socio-ecological model recognizes that there are societal or cultural high-level factors that create a climate that encourages or discourages behaviours. Broadly speaking, governments and the animal and public health communities create a climate that impacts willingness to report EID events. This process is operating at the level of nations, veterinarians, animal health care workers, and producers. Surveillance programs can serve to improve the relationship between veterinarians and government regulatory bodies [39]. The AVSN-VPS has generated information concerning the perspective veterinarians have on health-related events in the cattle population. This information has been shared between private cattle veterinarians and veterinarians at the ARD and has created a knowledge base around which to dialogue. Participants highlighted opportunities to enhance this relationship, in particular the need for diagnostic laboratory support guided by the outputs of the AVSN-VPS. The needs of consumers, producers, veterinarians, and the provincial and federal government could be well served were the ARD to utilize the willingness of veterinarians to participate in surveillance and participants’ recognition of the need for change within the veterinary profession. A collaborative effort between cattle veterinarians and veterinarians at the ARD to develop a government-supported diagnostic laboratory surveillance program that satisfied veterinarians’ desire for further diagnostic laboratory support, the requirement of the provincial and federal government to surveil for and report potential EIDs events as part of Canada’s membership in the OIE, and the public’s need to be assured of a safe food supply could enhance the relationship between cattle veterinarians and the ARD. This type of endeavour could be invaluable during future EID events, particularly as control of past events has required cooperation among producers, veterinarians, and multiple government agencies [41]. The CFIA should explore means of improving their relationship with cattle veterinarians as they are integral to detection of outbreaks of OIE-listed diseases and evidence of a healthy working relationship between the two parties from the perspective of participants was lacking.

### The Veterinary Perspective

Participants highlighted that at the time the interviews were conducted the beef industry and the cattle veterinary profession in Alberta were going through a period of significant change, and that the two are strongly linked. Previously, cattle veterinarians had an important role in performing technical procedures, such as caesareans, to treat animal disease conditions. They also had a role in implementing management programs that have decreased the burden of animal health conditions requiring veterinary intervention. Producers have learned alongside veterinarians and no longer require veterinarians to perform all the functions they did previously. Participants in this study identified this phenomenon and the need for the veterinary profession to change in response, though there were differences between participants in what those changes might need to be. From an EID standpoint, one avenue the cattle veterinary profession might consider exploring is increasing its emphasis on healthy animal populations, as opposed to animal populations that are simply free from disease: healthy animals are more resistant to infectious diseases [42], [43] and could serve to help mitigate the risk of future EID events in animal populations. If the model of private veterinary services to food-producing animals is going to persist, changes to the services provided by the veterinary profession are going to have to be economically relevant to producers.

Participants perceived that farming has historically attracted individuals that value independence and privacy. As a result there is inherent potential for conflict between producers and the need for improved government-driven EID surveillance. Future surveillance initiatives will need to consider this aspect of cattle production to encourage producer involvement and to help build an industry that attracts a future generation of farmers. Participants also highlighted that challenges to the beef industry in Alberta have made raising beef cattle less economically viable and that BSE in Canada has placed producers under considerable strain: producers fear not only a reportable or notifiable disease but the stigma that would come along with being ‘the guy in the community that’s got a positive’. Participants believed that producers were bearing much of the cost of surveillance and had yet to realize the benefits of surveillance programs initiated in part in response to the BSE crisis. These circumstances remain an ongoing challenge to surveillance: the negative consequences of an EID or reportable or notifiable disease are more tangible than the purported benefits associated with robust surveillance initiatives [36]. As surveillance serves the interests of producers, the food-producing industry, consumers, and the public [44], distributing the economic burden of surveillance among these parties is warranted. Though the cost of pathogen surveillance in animals is already distributed among these parties, the opinion expressed by participants suggests that further study is needed to ensure cost sharing is equitable.

The economic impact of delayed detection of future epidemics could be tremendous [45], [46]. Though the damage caused by delayed detection has been clearly demonstrated through retrospective analysis of previous outbreaks [35], these observations have been insufficient to motivate a global effort sufficient for early EID event detection and response [47]. A component of this issue is the relative lack of attention that has been paid to the social elements of EID surveillance. In order to be more effective, future surveillance initiatives need to incorporate an enhanced understanding of the human dimension of surveillance to encourage people closest to EID events to recognize, report, and respond.

### Conclusions

Diagnostic laboratory case submission by participants was biased toward cases in which multiple animals were affected and test results were of direct consequence to clinical case management. Participants also indicated that the expected level of disease varied between farms according to management practices. Broader economic factors, including the cost of diagnostics relative to the value of individual beef cattle and decreasing government support for laboratory diagnostics, limitations of diagnostic laboratory testing, and decreasing veterinary presence on farms, together translated into a decline in case submissions to diagnostic laboratories over time. Efforts to network diagnostic laboratories are unlikely to overcome this challenge to detection of animal disease threats, particularly if these threats occur sporadically or as a result of changes to trends in known endemic problems.

The responses from participants demonstrate that cattle veterinarians in Alberta are an underutilized resource in terms of EID surveillance: they have a perspective on cattle health and a relationship with producers that could prove critical to future EID event detection and response. In order for governments to realize this group’s potential for surveillance purposes there needs to be: adequate compensation for time and effort invested; generation of information that is clinically relevant; collaboration on surveillance system design, implementation, and evaluation; and due respect shown to the importance of the relationship between veterinarians and cattle producers. Governments face the added challenge of assuring producers that they will not disproportionately bear the social and economic costs of future EID events.
